# Educational outreach and collaborative care enhances physician's perceived knowledge about Developmental Coordination Disorder

**DOI:** 10.1186/1472-6963-8-21

**Published:** 2008-01-24

**Authors:** Robin Gaines, Cheryl Missiuna, Mary Egan, Jennifer McLean

**Affiliations:** 1 Children's Hospital of Eastern Ontario Research Institute, 401 Smyth Road, Ottawa, K1H 8L1, Ontario and School of Rehabilitation Sciences, University of Ottawa, Ontario, Canada; 2 School of Rehabilitation Science and *CanChild*, Centre for Childhood Disability Research, McMaster University, Hamilton, Ontario, Canada; 3 School of Rehabilitation Sciences, University of Ottawa, Ontario, Canada; 4 Department of Pediatrics, Dalhousie University, Halifax, Nova Scotia and IWK Health Centre, Halifax, Nova Scotia, Canada

## Abstract

**Background:**

Developmental Coordination Disorder (DCD) is a chronic neurodevelopmental condition that affects 5–6% of children. When not recognized and properly managed during the child's development, DCD can lead to academic failure, mental health problems and poor physical fitness. Physicians, working in collaboration with rehabilitation professionals, are in an excellent position to recognize and manage DCD. This study was designed to determine the feasibility and impact of an educational outreach and collaborative care model to improve chronic disease management of children with DCD.

**Methods:**

The intervention included educational outreach and collaborative care for children with suspected DCD. Physicians were educated by and worked with rehabilitation professionals from February 2005 to April 2006. Mixed methods evaluation approach documented the process and impact of the intervention.

**Results:**

Physicians: 750 primary care physicians from one major urban area and outlying regions were invited to participate; 147 physicians enrolled in the project. Children: 125 children were identified and referred with suspected DCD. The main outcome was improvement in knowledge and perceived skill of physicians concerning their ability to screen, diagnose and manage DCD. At baseline 91.1% of physicians were unaware of the diagnosis of DCD, and only 1.6% could diagnose condition. Post-intervention, 91% of participating physicians reported greater knowledge about DCD and 29.2% were able to diagnose DCD compared to 0.5% of non-participating physicians. 100% of physicians who participated in collaborative care indicated they would continue to use the project materials and resources and 59.4% reported they would recommend or share the materials with medical colleagues. In addition, 17.6% of physicians not formally enrolled in the project reported an increase in knowledge of DCD.

**Conclusion:**

Physicians receiving educational outreach visits significantly improved their knowledge about DCD and their ability to identify and diagnose children with this condition. Physicians who collaborated with occupational therapists in providing care reported more confidence in diagnosing children with DCD and were more likely to continue to use screening measures and to provide educational materials to families.

## Background

Developmental Coordination Disorder (DCD) is a chronic neurodevelopmental condition that affects 5–6% of children [1]. No generally accepted definition of this condition was available until 1989 [2]. The relatively recent inclusion of this diagnosis in the Diagnostic and Statistical Manual of Mental Disorders may explain the difficulty that many parents experience obtaining an accurate diagnosis when their children present with significant motor problems that impact on everyday activities [3].

Recognition of this disorder is critical. Due to their motor incoordination, these children are frustrated in schoolwork, self-care, sports and recreation. To the untrained, these difficulties often appear to be the result of immaturity, laziness, or uncooperative behavior [4,5]. When unrecognized and unmanaged, DCD can lead to long-term negative consequences including academic failure, [6,7] poor social relationships, [8,9] emotional difficulties, [10,11] mental health problems, [12] and poor physical fitness [13,14].

Physicians have ongoing contact with their young patients and are trusted by parents as the first resource for health care and referral to other providers. Therefore, physicians are in an optimal position to support families over time. Physicians can effectively manage DCD by recognizing and communicating a diagnosis to the family and then monitoring and supporting the family in the long term management of the child's healthcare needs. Physicians have the expertise to collect a detailed history, conduct a physical and neurological examination to rule out other causes of motor coordination. These steps are necessary in providing a differential diagnosis of DCD. However, at present, lack of physician knowledge about DCD is a major barrier to effective management of this chronic condition. To improve this knowledge gap, we designed and evaluated a demonstration project to enhance primary care physicians' ability to manage this under-recognized, but common childhood condition.

It is well-established that increasing health provider knowledge requires multi-faceted, interactive and repeated interventions [15-17]. Interventions such as educational outreach (personalized visits to a health care provider in his or her own setting) are particularly responsive to the specific information needs of the practitioner [18]. While research has demonstrated the effectiveness of educational outreach, the addition of collaborative care (in which another health professional provides specialized assessment and shares the process of supporting the family with the physician) may have promise for improving the management of chronic developmental disorders. Rehabilitation professionals, particularly occupational therapists (OTs) who have expertise in evaluating and enhancing motor-based functional activity, may be key members of such a collaborative health care team for physicians and children with DCD.

This demonstration project was conducted to determine the impact of a program, offering educational outreach and collaborative care, to improve identification and management of children with DCD by primary care physicians.

## Methods

### Study design

A mixed methods evaluation approach which employed pre- and post-project surveys, quantitative measures, questionnaires and focus groups was used to evaluate this demonstration project [19]. Approval for this study was granted by the Children's Hospital of Eastern Ontario Research Ethics Board.

### Participants

Community physicians, including community-based pediatricians and family medicine physicians, were recruited during information sessions using a faxed letter to the 750 primary care physicians practicing in the region. Physicians who joined the study were able to select from, and participate in, a number of educational outreach activities described below. Following education, participating physicians were able to refer children aged 4–12 years to the study for collaborative care if they suspected the child may have DCD, and had ruled out other potential explanations for the motor problems (e.g., head trauma, muscular dystrophy).

### Intervention

#### Educational outreach

A multi-faceted approach was used [16]. Educational materials for physicians were developed systematically, working with primary care physicians and representatives of the College of Family Physicians [20]. An inter-professional team including a developmental pediatrician, speech-language pathologist and psychologist provided informational support to an occupational therapist (OT) who worked in the community. The OT provided education to physicians in their offices using an array of materials that were developed for the project including: user-friendly, evidence-based written information presented in a binder; reminder folders which prompted physicians to carry out the stages of the screening process; tear-off interview guides; a parent-friendly waiting room advertisement, as well as a DVD presenting typical motor behaviour of children with and without DCD. Project information, sample video clips and materials were also made available electronically [21] (username: dcdpack; password: dcdchild). These materials are still available and continue to be used by physicians as a resource (please see Additional file 1 for details of physician educational materials).

The OT tailored the educational opportunities for each physician according to learning needs and style, background, interests, time availability and preference for group or individual meetings.

#### Collaborative care

Participating physicians were invited to apply their new knowledge in their practice to screen their patients and then refer any child suspected as having DCD to the OT for further evaluation. The OT administered the *Movement Assessment Battery for Children *[22], a test of motor impairment, and then interviewed the parent about the impact of the child's motor abilities on functional activities. Each child was screened using the *Kaufmann Brief Intelligence Test-2 *(K-BIT) [23] to rule out significant global developmental delay as the cause of motor difficulties. Following the assessment, the OT provided a summary of the results to the physician to assist him/her in determining whether a diagnosis of DCD was appropriate.

**Figure 1:**
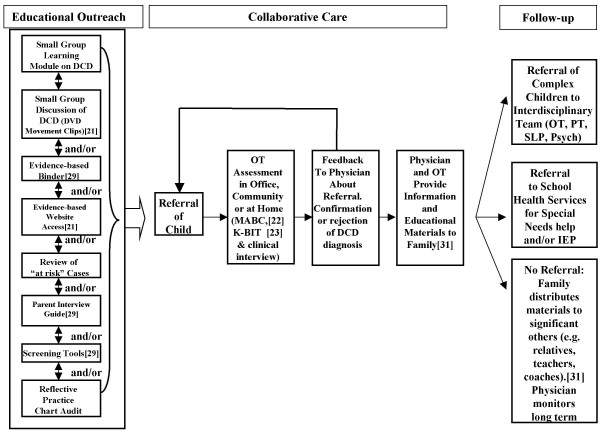
**Educational Outreach & Collaborative Care**. MABC = Movement Assessment Battery for Children; K-BIT = Kaufman Brief Intelligence Test; OT = Occupational Therapist; PT = Physiotherapist; Psych = Psychologist; SLP = Speech/Language Pathologist; IEP = Individualized Educational Plan

Tailoring services to the physician's needs, the OT assessed the child either with or without the physician present, provided feedback in written form and discussed the results with the physician. The OT and the physician then jointly presented the findings to the parents. A wide variety of educational materials was made available to parents at this time including handouts that could be shared with teachers, coaches, community leaders and other physicians. (See Additional file 1 for details of these family educational materials). Materials were available in English and French and were selected by the OT and physician based upon the child's identified needs. OT collaborative care services including conducting a clinical assessment with child and family, consultation with the physician and provision of feedback to the family, took between 3 and 4 hours per child in total. A physician's involvement in collaborative care including screening of the child's motor abilities, discussion with the family, consultation with the OT and provision of feedback took between 1 and 3 hours per child. The process of educational outreach and collaborative care is illustrated in Figure 1.

### Impact measurement

The impact of the demonstration project was evaluated in a number of ways. First, the number of physicians who chose to participate in the project, within the timeframe offered, is regarded as an indicator of the interest and need for educational outreach on this topic of DCD. Second, the number of children who were suspected of having DCD, referred to OT, received collaborative care and were given a diagnosis of DCD, is regarded as a measure of the effectiveness of the educational outreach.

To determine whether or not there was any change in physician awareness of DCD, a baseline survey of all physicians in the Ottawa region was used to establish their pre-project level of knowledge and perceived skill in diagnosing children with DCD. An identical post-project survey was conducted at project completion. In both surveys, physicians were asked to indicate one statement that best described their *knowledge *about DCD and one statement that best described their *skills *in recognizing and diagnosing a child with DCD. Responses were scored on a scale of 1 to 7, with 1 representing the least knowledge and skill and 7 representing the greatest knowledge and skill. In the second survey, participant physicians' responses were compared with responses from non-participating physicians throughout the region.

Participating physicians also completed two post-intervention questionnaires. In the first questionnaire they were asked questions about the perceived *usefulness *of the educational tools (e.g., screening activities, parent interview guide, family educational materials) and contribution of education and child-specific information from the OT. In the second questionnaire they were asked to indicate whether they planned to continue using the materials and/or share them with their colleagues.

Impact of the project on participating physicians was also examined qualitatively through *focus groups*. Only those physicians who had completed the educational outreach and had worked in collaborative care with the OT by the time of the focus group were invited to participate. Physicians were invited to a focus group according to their specialty (i.e. family medicine or paediatrics). All focus groups were audio-recorded and transcribed verbatim.

### Analysis

Quantitative results of the questionnaires are presented descriptively. Data from the questionnaire asking physicians to report on *usefulness *are summarized so that response scores of 1–4 are judged to be negative, and scores of 5–7 positive. Focus group findings were content-analyzed using methods recommended by Krueger and Casey [24].

## Results

Of the 750 physicians (678 family physicians and 72 community paediatricians) in the Ottawa region who were invited to participate, 147 physicians enrolled in the study over a 14 month period (see Table 1). Eighty four outreach visits were provided to these physicians by the OT for purely educational purposes: these included small group educational sessions and one-on-one discussions with physicians. In the majority of cases, these sessions occurred before the participating physicians referred children to the project. The median length of a 'face-to-face' visit with a physician was 45 minutes (range = 1 to 120 minutes).

**Table 1:** Physician participants

**PHYSICIANS (n = 147)**	
Male/Female	47(32%)/100(68%)
Community pediatricians	30(20.4%)
Family Physicians	117(79.6%)
Type of Primary Care Practice:	
Group	100(68%)
Sole practitioner or with partner	36(24.6%)
Not reported	11(7.4%)
Community-based practice	136(92.6%)
Community & hospital practice	11(7.4%)
Years in practice:	
<11 years	44 (29.9%)
11–20 years	57 (38.8%)
>20 years	35 (23.8%)
Not reported	11 (7.5%)
Number of children seen annually:	
<200 children/year	59 (40.1%)
200–500 children/year	28 (19.0%)
>500 children/year	30 (20.4%)
Don't know	10 (6.8%)
Not reported	20 (13.6%)

Sixty-four of the 147 physicians (44 family physicians and 20 community pediatricians) referred at least one child to the OT for assessment and collaborative care. Initially, physicians were limited to 3 referrals; however, as the study progressed, a few physicians asked if they could refer additional children and this was permitted. The number of children referred for collaborative care by each physician ranged from 1–5, mode = 1. A total of 125 children were referred and, of these children, 116 (92.8%) met the study inclusion criteria and were assessed by the OT. Children who were not seen were excluded due to age cut-off, previous diagnosis of other conditions (e.g., autism), and/or evidence of neurological conditions (e.g., seizure disorder). The age of children who received collaborative care ranged from 48 – 153 months (X = 96.3 months) and 87 (75%) were male.

Following assessment by the OT, 16 children (13.8%) were determined not to have motor delays substantial enough to warrant a diagnosis of DCD, according to study criteria. Cut-offs were based upon DSM IV diagnostic criteria [1], and are outlined in more detail elsewhere [25]. Another 12 children (10.3%) were determined to have motor impairment that was better explained by other conditions (orthopedic (1), trauma (3), and generalized delay (8)). The remaining 88 (76%) met research criteria for DCD; all but one were subsequently given a clinical diagnosis of DCD by their physician. In the one exception, the physician felt that environmental factors such as limited exposure to motor activities and family situation may have been the primary cause of the child's motor difficulties. The family did not return for consultation with the family physician, so further exploration of developmental versus environmental issues was not possible.

Pre-project *knowledge and skill *surveys were sent out to all 750 primary care physicians in the region. With a return rate of 25% (191 physicians), 91.1% of physicians reported that they were unaware of the condition of DCD and only 1.6% reported that they felt able to diagnose children with the condition. Post-project, the same survey was sent to all 750 physicians in the region, including physicians who had participated in the project and those who did not join the study. Two hundred and seventy-six questionnaires were returned for an overall regional return rate of 37%. The return rate for participating physicians was 61% (89/147) and 31% (187/603) for non-participating physicians. Table 2 depicts the results from the post-project questionnaire, asking physicians to describe their knowledge about DCD and their skill in making the DCD diagnosis. Results indicate that 91% of physicians who received educational outreach and responded to the questionnaire reported that they have *knowledge *about DCD in comparison with non-participating physicians in the same community among whom only 17.6% reported familiarity with the condition. In response to the perceived *skill *question, 41.6% of participating physicians reported an ability to diagnose children who had DCD, while only 3.2% of non-participating physicians believed that they could make this diagnosis.

**Table 2:** Knowledge and skills as reported by physician participants and physicians in the region, after project completion

**PHYSICIAN QUESTIONNAIRE**		
	Participating Physicians n = 89	Non-Participating Physicians n = 187

**KNOWLEDGE**		

Never heard/Limited Knowledge	8 (9%)	145 (77.5%)
Familiar with DCD	81 (91%)	33 (17.6%)
No response		9 (4.8%)

**SKILLS**		

Not able to recognize child with DCD	4 (4.5%)	45 (24.1%)
Observe motor skill difficulties, but do not discuss with parents	6 (6.7%)	41 (21.9%)
Can screen for motor difficulties but would refer to specialist	38 (42.7%)	82 (43.9%)
Able to diagnose DCD	37 (41.6%)	6 (3.2%)
No response	4 (4.5%)	13 (6.9%)

Results of the post-project questionnaire asking physicians to rate the *usefulness *of the diagnostic tools and collaborative care process are presented in Table 3. Questionnaire responses are reported from the 33 physicians who received educational outreach *and *participated in collaborative care with the OT and the 54 physicians who received educational outreach, but did not refer a child.

**Table 3:** Usefulness of project activities, as reported by physician participants*

**Evaluation of Intervention**	**Educational outreach and collaborative care** (N = 33). N and % reporting positive usefulness**	**Educational outreach only** (N = 54). N and % reporting positive usefulness**
How useful has the project been in helping you learn to *identify *children with DCD?	33 (100%)	28 (51.9%)
How useful are the DCD *screening activities *in your examination?	26 (78.8%)	23 (42.6%)
How useful is administering the *parent interview *guide?	25 (75.8%)	22 (40.7%)
How useful has the project been in helping you *diagnose *children with DCD?	32 (97%)	20 (37%)
How useful has it been to *share responsibility *for identification and management of a child in your practice with an OT?	31 (94.0%)	n/a

*Usefulness questionnaires were completed by 87/147 (59.2%) of participants

**Responses to each question were scored on a scale of 1 to 7 with 1 representing 'not very useful' and 7 representing 'highly useful'. Positive was determined to be scores of 5–7.

Responses to questions asking physicians about their plans for continued use of the project materials were received from 32 physicians who received educational outreach and collaborative care, and from 52 physicians who received educational outreach only. All (100%) of physicians answering this question (n = 32) who received both services indicated that they would continue to use project materials and resources, and 59.4% (n = 19) reported that they would recommend/share the materials with a medical colleague. Most (90.4%) (n = 47) of the 52 responding physicians who received educational outreach only indicated that they would continue to use the project materials and 28.8% (n = 15) reported that they would recommend the materials to their colleagues.

### Focus group results

Twenty four physicians (9 Family Physicians and 15 Pediatricians) who had completed both educational outreach and collaborative care at the time of the focus group were invited to attend. Four physicians attended the family medicine focus group and 13 physicians attended the paediatrician focus group. Physicians reported 'lack of availability' as their reason for not participating in the focus groups. Analysis of the transcripts indicated that both family physicians and pediatricians noted that they now had an increased awareness of the possible presence of DCD and had introduced regular screening techniques into their practices. These techniques ranged from routinely asking parents if they felt their children were "clumsy", observing the child taking off a shirt, or asking parents to complete a short questionnaire. As one pediatrician remarked, *"It seems that all these kids are in my practice, I just didn't identify them before"*.

Both groups of physicians reported appreciating the opportunity to have a more in-depth evaluation by the OT of children whom they had screened as having possible DCD. Many in the pediatrician group found this evaluation critical for children whose difficulties appeared marginal on screening. *"The ones you [identify on screening] are the obvious ones, but the ones that are in between, do they need it, do they not need it, where do you make the cut off point? You really need someone who can actually do that fine-tuning...We don't have time to do that."*

Both groups of physicians found the educational outreach valuable. Family physicians were enthusiastic about the tools for screening and the educational materials for families. For example, one family physician noted, *"I'm doing counseling, I'm telling the mom various different sports that might be better for them, where they succeed, where they may also develop some other skills...instead of setting them up for another failure. I think that's very important, and I've been doing that in the context of a physical." *Another family physician identified a role for herself in the context of public education and remarked that she was now sharing the information about DCD with adult patients and friends who are teachers.

## Discussion

The results of this study suggest that educational outreach and collaborative care provided within a primary care setting had a substantial impact on physicians' knowledge about children with DCD. There are very few examples in the literature of multidisciplinary or collaborative care approaches designed to increase the ability of primary care physicians to manage chronic childhood developmental disorders. In one recent study by Connor [26], primary care pediatricians were assisted by child psychiatrists to improve their evaluation and management of ADHD, childhood depression or anxiety disorders. A multidisciplinary/coordinated approach has also been reported for the management of individuals with Down Syndrome [27]. To the extent of our knowledge, there have been no other studies of the impact of a rehabilitation professional providing educational outreach in primary care settings to enhance physician knowledge and skills about a chronic childhood condition.

Increases in physician-reported comfort in managing children with common psychosocial and mental health problems, such as ADHD or social-emotional difficulties, have been shown to be positively related to receiving continuing medical education [28]. Primary care physicians have suggested that innovative programs improve their confidence and help modify their attitudes about the importance of childhood mental health problems [28]. Physician attitudes and confidence were not directly measured in our project; however, a substantial increase in reported knowledge and skill was found for participating physicians, as compared with physicians in the region who did not receive educational outreach. Of interest, physicians who worked directly with the OT in providing collaborative care for a particular child reported even greater knowledge and enhanced skill. In addition, these physicians reported that they were more likely to share the information with their colleagues. Active collaboration with another health professional, as an additional feature of the continuing education process, may be optimal for knowledge uptake about these types of conditions.

### Limitations of the study

Study limitations may restrict the generalizability of our results. There was a dramatic gain in perceived knowledge and skill among physicians who received these services compared to those who did not. It is possible that these participants may have represented a group of highly motivated physicians, who would have worked independently to develop their knowledge and skill when they encountered a diagnosis with which they were unfamiliar. However, it is unlikely that such self-selection bias accounted for all of the difference between physicians who received outreach and collaboration and those who did not, as resources about DCD had been available before this project began.

Second, the physicians who self-referred to this study may not be a representative sample. In this study, the majority of the physician participants (68%) were women. While the proportion of women in medicine is increasing, female physicians remain a minority in Ontario and among them a large proportion are relatively recently qualified. It is possible that female and more recently qualified physicians may be more open to collaborative practice and outreach education from other professionals.

While we are unable to make a definitive statement about the size of the effect of this intervention in other regions, this project demonstrates the feasibility and potential impact of outreach education and collaborative care with rehabilitation professionals to improve the management of children with this chronic condition.

## Conclusion

Ideally, best practice in service provision for children with chronic conditions is evidence-based. It can be difficult for primary care physicians to remain current about screening practices and management concerning all chronic childhood conditions. Rehabilitation professionals have a 'specialized' knowledge set that is often more focused in scope. Integration of these professionals into primary care settings allows for current knowledge about identification and management of chronic childhood conditions to be shared with physicians. The actual cost of implementing a model of care such as this is difficult to determine. Physicians who participated in this project were not reimbursed in any way for their time but joined the project in order to gain new knowledge. Comments raised during focus group sessions suggest that primary care physicians have an awareness of the presence of these children in their practices but may not have previously known how to respond to parents' concerns. Missiuna and colleagues [3] have previously demonstrated the high cost, to the family and to the healthcare system, of parents being referred by their physician to one specialist after another, seeking information that would help them understand the difficulties being experienced by their child with DCD. When the family physician is knowledgeable enough to respond and can work with an OT to provide collaborative care, it is probable that the needs of children and families would be met in a more timely and effective manner, and at a lower cost.

It is possible that the model of educational outreach and collaborative care used in this project may be applicable for improving physicians' identification and management of other chronic childhood conditions first identified in primary care settings. Autism, attention deficit hyperactivity disorder, chronic obesity, and specific language impairment are examples of conditions about which rehabilitation providers (speech/language pathologists, occupational therapists, physiotherapists, psychologists) may be able to share knowledge that would support physicians in their provision of high quality, evidence-based care. Further studies of educational outreach are needed to look at the types of information and patients who may be served by these professionals.

## Competing interests

The author(s) declare that they have no competing interests.

## Authors' contributions

All authors have read and approved the final manuscript.

RG had full access to all of the data in the study and takes responsibility for the integrity of the data and the accuracy of the data analysis.

Study concept and design: RG, CM.

Acquisition of data: RG.

Analysis and interpretation of data: RG, CM, ME, JMcL.

Drafting of the manuscript: RG, CM, ME, JMcL.

Critical revision of the manuscript for important intellectual content: RG, CM, ME, JMcL.

Statistical analysis: RG, CM, ME.

Obtained funding: RG, CM, ME, JMcL.

Administrative, technical, or material support: RG, CM, ME, JMcL.

Study supervision: RG, CM, ME, JMcL.

## Pre-publication history

The pre-publication history for this paper can be accessed here:



## Supplementary Material

Additional file 1 Physician and family educational materials. detailed description of materials and references for finding them. Click here for file
