# Supplementary material for: Educational outreach and collaborative care enhances physician's perceived knowledge about Developmental Coordination Disorder
Source: BMC Health Serv Res. 2008 Jan 24;8:21. doi: 10.1186/1472-6963-8-21 (PMC2254381; doi:10.1186/1472-6963-8-21)
Supplement: Additional file 1 — Physician and family educational materials. detailed description of materials and references for finding them. [file 1472-6963-8-21-S1.doc]

## Additional file 1 - Physician and family educational materials

Physician Educational Materials

- **Binder**: an attractive and user-friendly binder, the DCD Physician Allied health Collaboration Kit (DCD PACK) [29] was developed that provided succinct summaries of evidence-based information on the topics of: cues and clues to the presence of DCD-parenting challenges/parent and teacher concerns, differential diagnosis of DCD, assessing the child, managing and monitoring DCD, case study examples.
- **Website**: a website was developed that covered the same information as the binder, for physicians who prefer to access materials online [21] (to enter the web site use the following: username ‘dcdpack’ and password ‘dcdchild’ without the inverted commas)
- **Reminders**: laminated folders were produced which outlined the screening activities that the physician was to use; a tear-off pad was created with a short questionnaire for parents to complete; a laminate sheet of referral services in Ottawa
- **Waiting room advertisement**: a colorful flyer *(“Does your child have DCD?”)* [30] was created that presented briefly the key symptoms indicating the presence of DCD

Family Educational Materials

(*Please note: Development of some of the family materials was underway prior to this project. The project translated all materials, ensured that they were posted on the CanChild website* [31] *in English and French, and that primary care physicians knew how to access them*)

- **Parent booklet explaining DCD**: A booklet was developed that helps parents understand the characteristics of children with DCD and provides practical strategies for managing self-care issues in the home.
- **Parent flyers**: Flyers were developed for health promotion and prevention of secondary disability (e.g., participation in physical activity, school accommodations, annotated bibliography)
- **Flyers for teachers**: Flyers specific to each child’s grade level can be distributed by the family to the child’s teacher. Flyers were also provided for each subsequent grade so the family can manage the child over time.
